# Factor and Cluster Analysis for TCM Syndromes of Real-World Metabolic Syndrome at Different Age Stage

**DOI:** 10.1155/2020/7854325

**Published:** 2020-07-07

**Authors:** Shujie Xia, Jing Cai, Jianxin Chen, Xuejuan Lin, Shujiao Chen, Bizhen Gao, Candong Li

**Affiliations:** ^1^Syndrome Research Base of Traditional Chinese Medicine, Fujian University of Traditional Chinese Medicine, Fuzhou 350122, China; ^2^Fujian Key Laboratory of TCM Health State, Fuzhou 350122, China; ^3^College of Integrative Chinese and Western Medicine, Fujian University of Traditional Chinese Medicine, Fuzhou 350122, China; ^4^College of Traditional Chinese Medicine, Beijing University of Traditional Chinese Medicine, Beijing 100105, China; ^5^The Third People's Hospital of Fujian Province, Fuzhou 350108, China

## Abstract

**Background:**

Traditional Chinese medicine (TCM) has the characteristics of multitarget and overall regulation, which has certain advantages in preventing and treating of metabolic syndrome (MS). The aim of the present study was to evaluate the similarities and differences of TCM syndrome distribution in different age groups to promote the optimization of treatment strategies.

**Methods:**

This study was based on a real-world survey conducted in 3 hospitals in China. There are 1262 collected cases of MS meeting the inclusion criteria, which were divided into the young group, middle-aged group, and elderly group. Factor analysis (FA) was conducted for syndrome element extraction, and *K*-means cluster analysis was processed for syndrome type classification. Frequency analysis and Chi-square test were used to compare the syndrome characteristics of each group.

**Results:**

Common factors extracted were assigned to 18 syndrome elements including 11 nature syndrome elements and 7 location syndrome elements. Phlegm and dampness are the most frequent syndrome elements in general. Compared with the middle-aged group and elderly group, the young group has more obvious nature syndrome elements in heat and Qi deficiency, and location syndrome elements in the stomach. As for the middle-aged group, the frequency of location syndrome in kidney syndrome was higher than that in other groups. When it comes to the elderly group, it is shown that the symptoms of Yin deficiency and blood deficiency increase with age and the old patients may have more location syndrome elements in the lung and gallbladder.

**Conclusion:**

The TCM syndrome of MS is complex in that there may be the characteristics of deficiency and excess syndrome simultaneously. The main pathological factors of MS were phlegm and dampness. Besides, MS patients at different age periods may have their own syndrome distribution features. So, it is reasonable to adhere to the method of resolving phlegm and removing dampness and, at the same time, adopt the ways of clearing heat, promoting Qi, nourishing Yin, supplementing blood as well as regulating the liver, promoting the lung, transporting the spleen, nourishing the heart, and nourishing the kidney based on the syndrome feature of MS in different age stages.

## 1. Introduction

Metabolic syndrome (MS) is a metabolic disorder syndrome characterized by obesity, hyperglycemia, hypertension, dyslipidemia, and hyperuricemia, which are the pathological basis of cardiovascular and cerebrovascular diseases and diabetes [1]. Studies have shown that the incidence of MS increases with age, and the earlier the age of onset, the greater the risk of disease [2–4]. At present, the incidence of MS is 58.1% in the population over 60 years old in China, which seriously endangers people's health [5]. Traditional Chinese medicine(TCM) has the characteristics of multitarget, small side effects and overall regulation. There are many studies showing that the therapeutic effects of MS in TCM are fairly satisfying [6–8]. In TCM clinical practice, syndrome differentiation serves as the core of treatment for MS [9]. When differentiating a patient's syndrome pattern, the practitioner systematically collects comprehensive information about the presenting signs and symptoms by the four diagnostic methods of inspection, auscultation/smelling, interrogation, and palpating. Then, the collocated information is evaluated according to the TCM theory and clinical experience to identify the pathological characteristics of the current stage and differentiate the syndrome pattern. After that, TCM treatment will be applied in accordance with the conclusions drawn from the syndrome differentiation process.

Related studies focused on syndrome classification based on data mining methods. Various classification techniques, e.g., artificial neural network, naïve bayes, *k*-nearest neighbor, and multilabel learning were applied in quantitative syndrome analysis [10–13]. Better results were obtained for several important diseases, e.g., coronary heart disease, diabetes, and viral hepatitis. By introducing these data mining models, researchers can mine the objective syndrome feature distribution for diseases from some effective cases. Such feature distribution can be used to characterize the underlying relationship between the current disease status and its specific symptoms. There are some studies explore the overall syndrome distribution characteristics of MS or related diseases [14, 15]. However, few studies explored the underlying syndrome features in MS at different age stages. Even though it is the same disease, the treatment methods are different according to the syndrome characteristics of different age stages. So, it is of great significance to determine the syndrome features of MS at different age stages from effective clinical cases.

In this study, we use the multivariate statistical method to explore the distribution feature of TCM syndrome in MS patients at different age stages who really need a physical examination or TCM treatment. First, factor analysis (FA) was applied to reduce the dimensionality of a large number of TCM symptoms and signs, by which these TCM variables can be remodeled as linear combinations of a smaller number of underlying factors. Second, according to the underlying factors, i.e., common factors, the TCM syndrome elements were further extracted in combination with professional knowledge. Third, the distribution of TCM syndrome elements in different age groups was compared and analyzed based on factor scores. Then, the common factor scores of each patient were included as variables in the cluster analysis, which could find the potential combination rule of each factor and then classify each sample. Lastly, we summarized the main TCM syndromes types contained in each category and analyzed the distribution feature of syndrome types in different age groups. In this way, better TCM syndrome features can provide objective diagnostic evidence for clinical doctors to improve the accuracy of syndrome diagnosis and efficacy of clinical treatment for MS.

This paper consists of the following sections. Materials and Methods introduce case selection and analysis methods. Results show the syndrome distribution outcomes of MS in different age groups.

Discussion and conclusion analyze the reasons for the difference of syndrome distribution in different groups, and the limitations of this research and future works are also discussed.

## 2. Materials and Methods

### 2.1. Study Design and Participants

The inpatients and outpatients with MS were selected in the Second People's Hospital Affiliated to the Fujian University of TCM, the Third People's Hospital Affiliated to the Fujian University of TCM, the Fuzhou Second Hospital, and the Jinjiang Hospital of TCM from November 2013 to October 2019. The participants were divided into three groups according to the following criteria: young group (YG, 18–39 years old), middle-aged group (MG, 40–59 years old), and elderly group (EG, 60 years old and above). All participants signed consent forms. Ethics approval for the present study was given by the Medical Ethics Committee of the Fujian University of Traditional Chinese Medicine.

### 2.2. Sample Size Estimation

According to the number of four diagnostic information items that may appear in MS patients of all age groups and the principle of “the number of samples must be 5–10 times the number of variables,” the sample size was calculated considering the 10% missing rate. In this study, there were 45 symptom variables with a frequency of more than 10%, to ensure the accuracy of the study to the greatest extent, and the total sample size of the study was 1262.

### 2.3. Diagnostic Criteria

Diagnostic criteria of Western medicine were as follows: according to the MS diagnostic criteria issued by the IDF, AHA, and Diabetes Society of the Chinese Medical Association, the diagnosis can be made if the following 3 items or more are met: (1) abdominal obesity (waist circumference: male ≥90 cm and female ≥85 cm); (2) hyperglycemia: FPG ≥ 6.1 mmol/L or 2 hPG ≥ 7.8 mmol/L and/or diagnosed as diabetes; (3) hypertension: BP ≥ 130/85 mmHg and/or diagnosed as hypertension; (4) TG ≥ 1.70 mmol/L; and (5) HDL-C < 1.04 mmol/L. TCM syndrome element and type diagnosis standard was as follows: according to the *Syndrome Part of TCM Clinical Diagnosis and Treatment Terminology* (the National Standard of the People's Republic of China) [16], *TCM Diagnostics* [17], *Syndrome Elements Differentiation* [18] and combined with the expert consultation, the classification specification of syndrome patterns was formulated.

### 2.4. Inclusion and Exclusion Criteria

Inclusion criteria of the patients are (1) patients who meet the diagnostic criteria of MS; (2) patients who are given the informed consent; and (3) patients' age range from 18 to 75.

Exclusion criteria are (1) patients with mental diseases or other severe diseases; (2) patients who could not express their feelings clearly; and (3) patients who refuse to participate in our study or without informed consent.

### 2.5. Analysis Method

According to the “syndrome differentiation significance of 600 common symptoms” and the MS common symptom standard in the *Guideline of Clinical Research of TCM New Drugs*, the four diagnosis information collection scale is established. The symptoms and signs were classified as none, mild, moderate, and severe with 0, 1, 2, and 3 points, respectively. For symptoms and signs that are difficult to be graded, they were classified into nonoccurrence and occurrence with 0 and 1 points, respectively.

**Figure 1:**
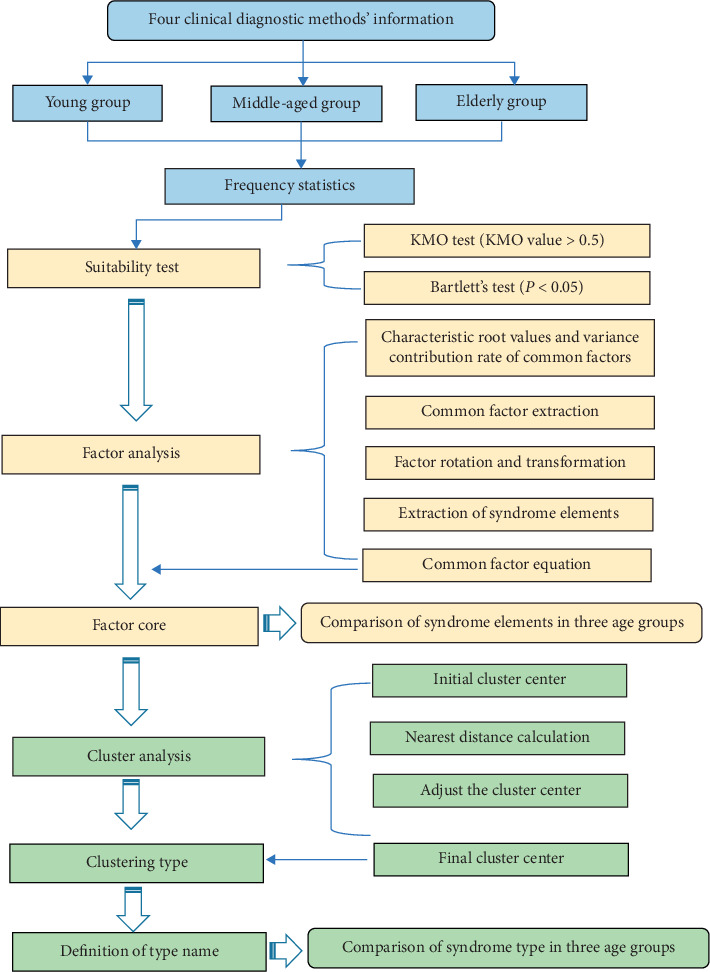
Flowchart of factor and cluster analysis-based MS syndrome pattern extraction. First, TCM clinical information of MS patients in different age groups was collected. Then, the collected variables were dimensionally reduced by factor analysis, and the main syndrome elements of each group were extracted. Finally, cluster analysis was used to classify the syndrome elements, so as to further summarize the distribution characteristics of MS syndrome types.

SPSS 24.0 software was used for statistical processing and analysis. Factor analysis was processed by the KMO test, Bartlett spherical test, and principal component analysis, and then the distribution of nature and location syndrome elements in different age groups was obtained. On the basis of factor analysis, the K-means clustering method was used to analyze the distribution of syndrome types in different age groups (Figure 1). The Chi-square test was used for the comparison of counting data, and the Bonferroni method was used for the comparison between two groups to correct the inspection level. The corrected inspection level *α* = (0.05/3) = 0.0167. All data were entered, counted, and processed by professionals.

### 2.6. Quality Control

The study was carried out with strict quality control. All investigators were specialized in TCM or integrated Chinese and Western medicine and trained in the standard operating procedures for the study. Each study participant was examined and followed up by at least two resident physicians or graduate students who filled in the CRFs. At least two senior staff physicians supervised the interview sessions to ensure consistency and authenticity of data collection to reduce measurement bias.

## 3. Result

### 3.1. General Result

In this study, 1262 cases of MS were collected (Table 1), including 554 males (43.90%) and 708 females (56.10%), with an average age of 51 years. There were 209 cases (16.56%) in the young group, 131 males and 78 females, with an average age of 33 years. There were 740 cases (58.64%) in the middle-aged group, 299 males and 441 females, with an average age of 50 years. There were 313 patients (24.80%) in the elderly group, 124 males and 189 females, with an average age of 65 years.

### 3.2. Frequency Analysis of Four Diagnostic Information

The four diagnosis information of MS patients in different age groups was tabulated based on the distribution frequency. Each group screened the first 20 symptoms and signs (Table 2).

### 3.3. Factor Analysis of Syndrome Elements

#### 3.3.1. Suitability Test

The KMO test and Bartlett's test of sphericity were used to evaluate the suitability of collocated diagnostic variables for factor analysis. The Kaiser–Meyer–Olkin (KMO) test assesses the partial correlation between variables, and if the KMO value is > 0.5, the variable will be more suitable for factor analysis. In addition, the closer the KMO value is to 1, the stronger the correlation is between variables. Bartlett's test of sphericity assesses the null hypothesis and whether the correlation matrix is rejected as a unit matrix. Only when the variables are relatively nonindependent (*P* < 0.05), they can be used for factor analysis.

In this study, the KMO value of the partial correlation of variables was 0.627 > 0.5, indicating a certain degree of partial correlation between variables and factor analysis could be carried out. The approximate Chi-square value of Bartlett's test of sphericity was 8403.438, and *P* < 0.001, indicating a strong correlation between variables and rejection of the hypothesis of independence of variables. So, the variables could be applied to factor analysis.

### 3.4. Extraction Factors

**Figure 2:**
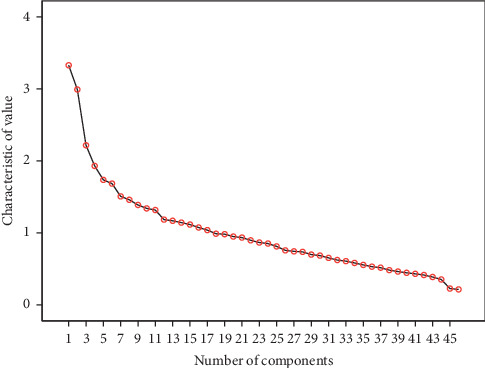
Scree plot of the characteristic root value of common factors. The number of common factors is shown on the *x*-axis and the characteristic values on the *y*-axis. Each node represents a common factor, which is arranged according to its characteristic value.

Characteristic root value is an index to evaluate the influence of the extracted common factors, which indicates how much the information of the original variables a common factor can explain. The cumulative variance contribution rate (CVCR) is the accumulation of variance contribution rate of the first *N* common factors, which shows the proportion of information of the first *N* common factors is covered. A total of 45 variables of symptoms and signs with a frequency of more than 10% are included in the factor analysis. The principal component analysis was applied to extract common factors. The characteristic root values of the first 19 common factors were no less than 1, and their CVCR reached 62.06%. From the scree plot (Figure 2), we can see the relevance of common factors and characteristic root values. The scatter locations of the first 19 common factors were steep, which means the first 19 common factors bear the most information in this research.

### 3.5. Factor Rotation and Transformation

The purpose of factor rotation is to allow the factor load the absolute value of the new common factor for each of the variables to polarize to 0 or 1. It can facilitate the naming and interpretation of the main factors in this study. After the maximum rotation of variance and setting 0.3 as the threshold value of the load coefficient, each common factor was allocated to the most relevant index. Based on the results of factor rotation and combined with professional knowledge, we summarized 18 syndrome elements (Table 3), including 11 nature syndrome elements and 7 location syndrome elements. The nature syndrome elements include phlegm, dampness, heat, cold, Qi stagnation, blood stasis, Yang hyperactivity, Yin deficiency, Yang deficiency, blood deficiency, and Qi deficiency, respectively. The location syndrome elements include the heart, lung, liver, spleen, kidney, stomach, and gallbladder.

### 3.6. Common Factor Equation for Syndrome Elements

Through the common factor equation, the scores of each patient on each of the 19 common factors were obtained. Then, the distribution of the syndrome elements in each group was analyzed according to the highest score. There were obvious differences in the overall distribution of nature and location syndrome elements in the three different age groups (*P* < 0.001) by the *X*^2^ test.

**Figure 3:**
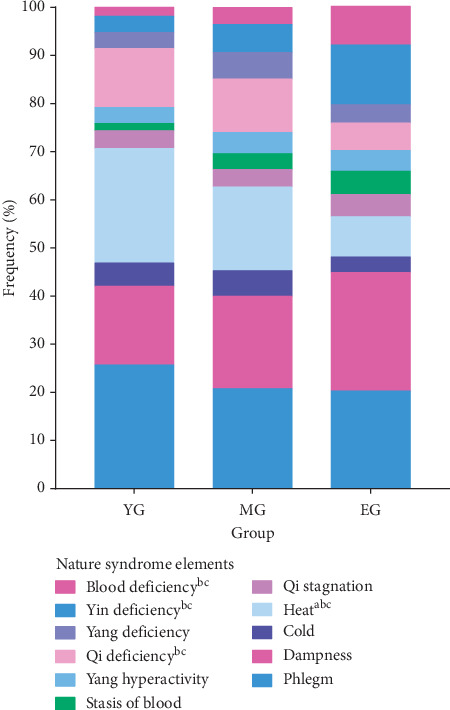
Distribution of nature syndrome in different age groups of MS patients. The nature syndrome element distribution of blood deficiency, Yin deficiency, Qi deficiency, and heat was obviously different among three age groups. ^a^A significant difference between the young and the middle-aged groups; ^b^a significant difference between the young and the elderly groups; ^c^a significant difference between the elderly and the middle-aged groups.

In the aspect of nature syndrome elements (Table 4, Figure 3), the top three frequencies in the young group were phlegm, heat, and dampness syndrome; in the middle-aged group, the top three frequencies were phlegm, dampness, and heat syndrome; and in the elderly group, the top three frequencies were dampness, phlegm, and Yin deficiency syndrome. Specifically, there were some significant differences in each group. In the elderly group, the frequency of blood deficiency and Yin deficiency syndrome was obviously higher than that in the other two groups, while the frequency of Qi deficiency syndrome was lower than that in the other groups. In the young group, the frequency of heat syndrome was obviously higher than that in the other two groups.

**Figure 4:**
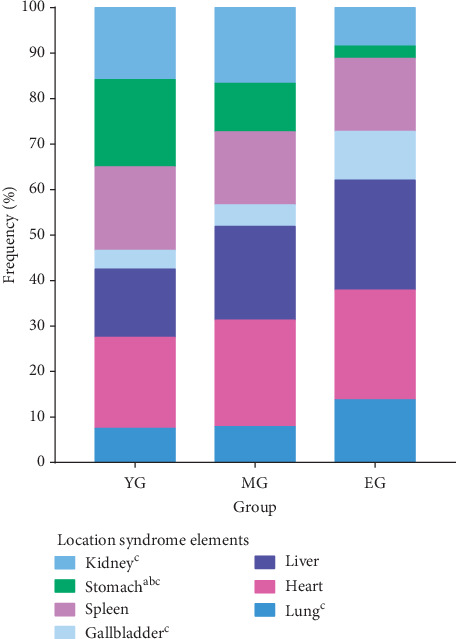
Distribution of location syndrome in different age groups of MS patients. The location syndrome distribution of the kidney, stomach, gallbladder, and lung was obviously different among three age groups.

In the aspect of location syndrome elements (Table 5, Figure 4), the top three frequencies in the young group were heart, stomach, and spleen syndrome; in the middle-aged group, the top three frequencies were heart, liver, and kidney syndrome; and in the elderly group, the top three frequencies were liver and heart, spleen, and lung syndrome. There are also some specific significant differences among these groups. For the young group, the frequency of stomach syndrome was obviously higher than that in the other two groups. For the middle-aged group, the frequency of kidney syndrome was obviously higher than that in the elderly group. For the elderly group, the frequency of lung, gallbladder, and kidney syndrome was higher than that in the middle-aged group.

### 3.7. Cluster Analysis of MS Syndrome Type

Scores of 19 common factors in each age group were taken as variables in the *K*-means cluster analysis. Then, those variables were clustered to 5 final cluster centers. It can be seen from the ANOVA table (Table 6) that almost all common factors contribute to this clustering with *P* < 0.001 (except for F3). The top five common factors owning the largest contribution are F2, F17, F8, F18, and F11. The CVCR of the combined factors was 20.86%, 15.18%, 10.54%, 10.73%, and 6.90%, respectively (Table 7).

**Figure 5:**
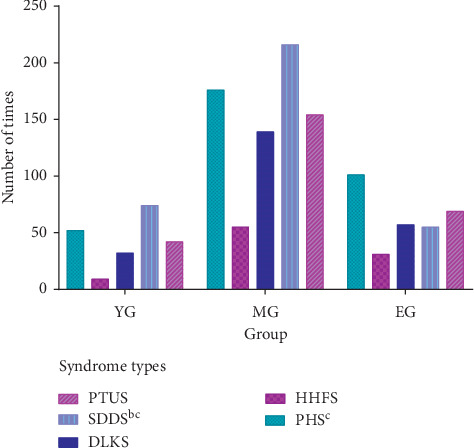
Distribution of syndrome types in different age groups of MS. The distribution of spleen deficiency and dampness syndrome and phlegm heat syndrome was obviously different among three age groups. PHS: phlegm heat syndrome; HHFS: hyperactivity of heart fire syndrome; SDDS: spleen deficiency and dampness syndrome; DLKS: deficiency of liver and kidney syndrome; PTUS: phlegm turbid upper syndrome.

Combined the cluster results with the expert's suggestions and the relevant standards of MS diagnosis in TCM, all samples were classified into five types: phlegm heat syndrome (PHS), hyperactivity of heart fire syndrome (HHFS), spleen deficiency and dampness syndrome (SDDS), deficiency of liver and kidney syndrome (DLKS), and phlegm turbid upper syndrome (PTUS). There was a significant difference in the overall distribution of syndrome types among the three groups by the X^2^ test (Figure 5). The top 3 frequencies of syndrome types in the three groups are spleen deficiency and dampness syndrome, phlegm and heat syndrome, and phlegm and turbid upper syndrome. The frequency of spleen deficiency and dampness syndrome in the young group was obviously higher than that in the old group. The frequency of phlegm heat syndrome in the elderly group was obviously higher than that in the middle-aged group.

## 4. Discussion

### 4.1. Multivariate Statistical Method and TCM Syndrome Differentiation

Syndrome differentiation and treatment is the essence of TCM [19]. The information collected from the four diagnostic methods (inspection, auscultation/smelling, interrogation, and palpating) is an important basis for the diagnosis and treatment of TCM. However, the TCM syndrome information has the characteristics of subjective, complex, multidimensional, multilevel, and so on, which increases the difficulty in clinical and scientific research of TCM syndrome. Factor analysis is a statistical method to find out the limited unobservable potential variables, i.e., common factors that dominate the relationship from the internal dependence of the correlation matrix of the original variables, and then explain the correlation or covariance relationship between the original variables [20, 21]. Cluster analysis is used to find a kind of statistics that can objectively reflect the affinity between things and then classify things according to these statistics and some classification criteria [21], which aim at making the differences within each category as small as possible, and the differences between categories as large as possible. In TCM, the syndrome cannot be observed directly but can be reflected by a series of symptoms and signs that may potentially be related. The basic unit of TCM syndrome is the syndrome element, including the nature and location syndrome elements, which is similar to the common factor. Factor analysis can be used to find the potential relationship behind the four diagnosis information of TCM, namely, the common factors (syndrome elements). The syndrome type is composed of different nature or location syndrome elements by certain rules, which is also similar to cluster analysis that can be used to find the potential combination rules from various syndrome elements and then classify each sample into a certain syndrome type.

### 4.2. The Similarities of Syndrome Distribution in MS Patient

The occurrence and development of MS are closely related to age. Traditional Chinese medicine emphasizes “adjusting measures to individual conditions” and “treating the same disease with different methods.” Despite having the same disease, patients of different ages may have different syndrome characteristics under the basic pathogenesis. Only by understanding the similarities and differences of diseases in different populations, the therapeutic effect can be improved. In this study, the common symptoms of MS, including thirst, dry throat, bitter taste, dizziness, chest distress, tiredness, flustered, impatience and irritability, lack of sleep, lumbago, blurred vision, fat body, slippery pulse or thin pulse, and white or yellow greasy coating, revealed the basic pathogenesis of excessiveness complicated with deficiency syndrome. The symptoms such as spitting, impatience, irritability, and slippery pulse indicate excess syndrome, and the dizziness, blurred vision, and tiredness suggest deficiency syndrome. Moreover, phlegm and dampness are the main pathological features in all age groups.

In clinical practice, it is found that most of the patients with metabolic syndrome are obese. According to the TCM theory, fat people are more likely to develop a phlegm-dampness constitution, so they have symptoms such as spitting, feeling body heavy and trapped, and greasy tongue coating. While phlegm-dampness is prone to hurting the spleen and hindering the movement of Qi and blood, so the patient may have symptoms such as epigastric distention, lassitude and weakness, spontaneous sweating, and dizziness. With diseases lasting for a long time, Qi stagnation and blood stasis will turn out, so symptoms such as bitter taste, impatience and irritability, purple lips, and wiry pulse can be found. Moreover, the phlegm-dampness can turn into heat with time going by, and pathogenic heat can also disturb the heart, which will induce symptoms of thirst, upset, fidgety, dreaminess, insomnia, and so on. When phlegm and blood stasis combine, MS would further develop and the symptoms of viscera deficiency appear, such as blurred vision, palpitation, waist and knee soft, and often loose stools. So, on the basis of statistical analysis and professional knowledge, we ended up with five syndrome types, including phlegm heat syndrome, hyperactivity of heart fire syndrome, spleen deficiency and dampness syndrome, deficiency of liver and kidney syndrome, and phlegm turbid upper syndrome.

### 4.3. The Differences of Syndrome Distribution in Different Age Groups of MS

Based on the commonness of MS, there are also some different characteristics among the young, middle-aged, and elderly group. Compared with the middle-aged group and elderly group, the young group has more obvious nature syndrome elements in heat and Qi deficiency, and location syndrome element in the stomach. It may be related to that the young people are just at the time of excess blood and Qi, and the excess of Qi can lead to heat syndrome, just like the famous TCM doctor Danxi Zhu said, “excess of Qi is fire.” So, young patients are more likely to have the heat syndrome compared to old patients. Heat evil is more likely to consume body fluid and Qi. Besides, an unhealthy diet style may easily damage Qi in spleen, so the young patients also have a higher frequency in Qi deficiency. As for the middle-aged group, the most obvious problem is that the frequency in kidney syndrome was higher than that in other groups. This may be because most patients in the middle-aged group were female patients and about 50 years old. At this time, they are just in perimenopause and appear more kidney deficiency problems [22]. When it comes to the elderly group, the mechanisms of MS and its syndrome features turn out to be more complicated. According to the results, it is shown that the symptoms of Yin deficiency and blood deficiency increase with age and those old patients may have more location syndrome elements in the lung and gallbladder. So, we can also find that the elderly people have a higher frequency of symptoms such as dizziness, bitter taste, spitting, and dry throat. The results of cluster analysis show the relative difference is not big, but from it, we also find that the young and middle-aged groups have a higher frequency in spleen deficiency and dampness syndrome and phlegm heat syndrome, while the elderly group has a higher frequency in phlegm heat syndrome and phlegm turbid upper syndrome.

### 4.4. Deficiency and Prospect

This research is based on the real-world investigation, and the results basically conform to the syndrome distribution feature of MS in the clinic. However, there are also some differences with the previous research, which may be affected by several factors. First of all, there are some differences in the sample size of the three age groups in this study, which may have something to do with disease characteristics. Secondly, the regional environment can also affect the study results. These cases were collected in Fujian, where the climate is mostly humid and heat, which may have an impact on the frequency statistics of dampness and heat syndrome in this study. In addition, due to the limitation of statistical methods, there are some deviation indexes in the classification results, which are slightly different from other indexes in the same category.

However, in a word, factor and cluster analysis methods in this study were processed according to the internal laws of the data themselves, which reduced the errors caused by subjective judgment and revealed the syndrome characteristics of MS in different age groups from different perspectives. In this way, it can provide a certain basis for the quantitative and qualitative syndrome differentiation and treatment of MS. In future research, a large-scale and multicenter epidemiological investigation can be combined. Also, the collection process of four diagnosis information needs to be more standardized and better-optimized analysis models are supposed to make it more suitable for the clinical characteristics of TCM.

## 5. Conclusion

From the above analysis, we can find that the TCM syndrome of MS is complex and there may have some syndrome elements that appeared simultaneously. However, it is not hard to find that the main pathological factors in MS were inseparable from phlegm and dampness. Besides, MS patients at different age periods have their own syndrome distribution characteristics. Therefore, in the clinical diagnosis and treatment process, we are supposed to focus on the syndrome feature in different age stages and give consideration to the primary and secondary aspects as well as the deficiency and excess aspects. So, it is reasonable to adhere to the method of resolving phlegm and removing dampness and, at the same time, adopt the ways of clearing heat, promoting Qi, nourishing Yin, supplementing blood as well as regulating liver, promoting lung, transporting spleen, nourishing heart, and nourishing kidney based on the syndrome feature of MS in different age stages.

## Figures and Tables

**Table 1:** Age and sex distribution of MS patients in three groups.

Group	Average age	Male		Female		Total	
		*N*	%	*N*	%	*N*	%
Young group	33	131	62.68	78	37.32	209	16.56
Middle-aged group	50	299	40.41	441	59.59	740	58.64
Elderly group	65	124	39.62	189	60.38	313	24.80
Total	51	554	43.90	708	56.10	1262	

**Table 2:** Frequency statistics of four diagnostic information.

Ranking	Young group			Middle-aged group			Elderly group		
	Variables	*N*	%	Variables	*N*	%	Variables	*N*	%
1	Wiry pulse	89	42.58	Wiry pulse	378	51.08	Wiry pulse	197	62.94
2	Slippery pulse	87	41.63	Thirsty	339	45.81	Thirsty	151	48.24
3	Thirsty	85	40.67	Greasy tongue coating	287	38.78	Greasy tongue coating	131	41.85
4	Greasy tongue coating	83	39.71	Slippery pulse	281	37.97	Slippery pulse	120	38.34
5	Pulse sinking	70	33.49	Bitter taste	225	30.41	Dizzy	119	38.02
6	Tooth-marked tongue	59	28.23	Dizzy	217	29.32	Bitter taste	118	37.70
7	Thready pulse	57	27.27	Acid heavy pain	211	28.51	Thready pulse	98	31.31
8	Pink tongue	54	25.84	Pulse sinking	200	27.03	Acid heavy pain	95	30.35
9	Thin white tongue coating	53	25.36	White tongue coating	191	25.81	Pharynx trunk	87	27.80
10	Yellow tongue coating	52	24.88	Pink tongue	188	25.41	Yellow tongue coating	81	25.88
11	Bitter taste	47	22.49	Yellow tongue coating	180	24.32	Chest tightness	77	24.60
12	Fat and sweet	47	22.49	Thready pulse	179	24.19	Sleep disturbance.	77	24.60
13	White tongue coating	46	22.01	Tooth-marked tongue	179	24.19	Spitting	76	24.28
14	Impatient and irritable	44	21.05	Thin white tongue coating	179	24.19	Blurred vision	72	23.00
15	Thirsty to drink cold	44	21.05	Lumbago	175	23.65	Insomnia	71	22.68
16	Tiredness and fatigue	43	20.57	Tiredness and fatigue	173	23.38	Pulse sinking	66	21.09
17	Addicted to spicy food	42	20.10	Fat and sweet	161	21.76	Rapid pulse	65	20.77
18	Body fat	41	19.62	Insomnia	157	21.22	Tooth-marked tongue	64	20.45
19	Acid heavy pain	41	19.62	Chest tightness	153	20.68	Lumbago	64	20.45
20	Rapid pulse	40	19.14	Blurred vision	148	20.00	Thick sputum	64	20.45

**Table 3:** Syndrome elements and the target index represented by common factors.

Common factor	Four diagnostic variables^*∗*^	Syndrome elements	
		Nature	Location
F1	Spitting 0.776, thick phlegm 0.760, sleep disturbance 0.359, greasy tongue coating 0.396, wiry pulse 0.314, heavy body trapped 0.422	Phlegm, dampness	Lung, spleen
F2	Upset 0.796, dysphoria 0.775, impatient and irritable 0.584	Yang hyperactivity	Heart, liver
F3	Yellow tongue coating 0.813, thick tongue coating 0.652, smoking 0.317	Phlegm, heat	—
F4	Blurred vision 0.685, dim eyesight 0.576, dizziness 0.563	Blood deficiency	Liver
F5	White tongue coating 0.804, greasy tongue coating 0.600, fatigue 0.309	Cold, dampness, Qi deficiency	—
F6	Flustered 0.740, palpitation 0.673, chest distress 0.539	—	Heart
F7	Lumbago 0.812, acid heavy pain 0.801	Dampness	Kidney
F8	Wiry pulse 0.654, lip purple 0.446, dry throat 0.355, smoking 0.309	Blood stasis, Yin deficiency	Liver
F9	Thirsty to drink heat 0.332, thirsty 0.745, pharynx trunk 0.619, bitter taste 0.475	Yin deficiency	Gallbladder
F10	High fat diet 0.675, addicted to spicy food 0.660, smoking 0.522	Phlegm, heat	Stomach
F11	Epigastric distention 0.664, heavy body trapped 0.486	Stagnation of Qi, dampness	Spleen
F12	Body fat 0.798, pink tongue 0.713	Sputum	—
F13	Sleep disturbance 0.350, dreaminess 0.767, insomnia 0.626	—	Heart
F14	Thirsty to drink heat 0.371, slippery pulse 0.535	Dampness	—
F15	Pulse sink 0.800	In	—
F16	Spontaneous sweating 0.802, thirsty to drink cold 0.475	Deficiency of Qi and heat	—
F17	Thin white tongue coating 0.585	Surface	—
F18	Constant fear of cold 0.768	Yang deficiency	—
F19	Waist and knees are soft and sour 0.611, frequent loose stools 0.569	—	Spleen, Kidney

^*∗*^Values in this table are results of the factor load matrix after rotation transformation.

**Table 4:** Distribution of nature syndrome in different age groups of MS patients.

Nature syndrome	Young group		Middle-aged group		Elderly group		Total	
	*N*	%	*N*	%	*N*	%	*N*	%
Phlegm	69	33.01	192	25.95	75	23.96	336	26.62
Dampness	44	21.05	178	24.05	91	29.07	313	24.80
Cold	13	6.22	49	6.62	12	3.83	74	5.86
Heat^abc^	64	30.62	161	21.76	31	9.90	256	20.29
Qi stagnation	10	4.78	34	4.59	17	5.43	61	4.83
Stasis of blood	4	1.91	30	4.05	18	5.75	52	4.12
Yang hyperactivity	9	4.31	41	5.54	16	5.11	66	5.23
Qi deficiency^bc^	33	15.79	103	13.92	21	6.71	157	12.44
Yang deficiency	9	4.31	51	6.89	14	4.47	74	5.86
Yin deficiency^bc^	9	4.31	54	7.30	46	14.70	109	8.64
Blood deficiency^bc^	5	2.39	33	4.46	29	9.27	67	5.31

^a^A significant difference between the young group and the middle-aged group. ^b^A significant difference between the young group and the elderly group. ^c^A significant difference between the elderly group and the middle-aged group.

**Table 5:** Distribution of location syndrome in different age groups of MS patients.

Location syndrome	Young group		Middle-aged group		Elderly group		Total	
	*N*	%	*N*	%	*N*	%	*N*	%
Lung^c^	9	4.31	40	5.41	36	11.50	85	6.74
Heart	24	11.48	118	15.95	63	20.13	205	16.24
Liver	18	8.61	104	14.05	63	20.13	185	14.66
Gallbladder^c^	5	2.39	24	3.24	28	8.95	57	4.52
Spleen	22	10.53	81	10.95	42	13.42	145	11.49
Stomach^abc^	23	11.00	54	7.30	7	2.24	84	6.66
Kidney^c^	19	9.09	84	11.35	22	7.03	125	9.90

**Table 6:** ANOVA.

Factor variables	Cluster		Error		*F*	Sig.
	Ms	Df	Ms	Df		
F1	40.616	4	0.874	1257	46.475	0.000
F2	194.800	4	0.383	1257	508.229	0.000
F3	2.090	4	0.997	1257	2.097	0.079
F4	28.403	4	0.913	1257	31.117	0.000
F5	22.410	4	0.932	1257	24.048	0.000
F6	5.472	4	0.986	1257	5.551	0.000
F7	31.322	4	0.904	1257	34.667	0.000
F8	58.548	4	0.817	1257	71.673	0.000
F9	19.032	4	0.943	1257	20.190	0.000
F10	11.051	4	0.968	1257	11.416	0.000
F11	51.446	4	0.839	1257	61.283	0.000
F12	6.060	4	0.984	1257	6.160	0.000
F13	6.639	4	0.982	1257	6.761	0.000
F14	15.807	4	0.953	1257	16.589	0.000
F15	6.494	4	0.983	1257	6.610	0.000
F16	44.689	4	0.861	1257	51.905	0.000
F17	94.035	4	0.704	1257	133.584	0.000
F18	53.126	4	0.834	1257	63.690	0.000
F19	26.020	4	0.920	1257	28.271	0.000

Df: degree of freedom; Ms: mean square.

**Table 7:** The factor constitution of syndrome classification.

Cluster	Factor combination	CVCR (%)	Syndrome type	Main signs and symptoms
1	*F*1 + F3 + *F*6 + F7 + F12	20.86	PHS	Spitting, phlegm is thick, sleep disturbance, heavy body trapped, smoking, flustered, palpitation, chest distress, acid heavy pain, obesity, thick or greasy tongue coating, yellow coating, wiry pulse, etc.
2	*F*2 + F13	10.73	HHFS	Upset, fidgety, impatient and irritable, sleep disturbance, dreaminess, insomnia, etc.
3	*F*8 + F9 + F11 + F14 + F17 + F19	15.18	DLKS	Thirsty, pharynx trunk, bitter taste, thirsty to drink heat, smoking, epigastric distention, heavy body trapped, waist and knees are soft and sour, frequent loose stools, wiry or slippery pulse, etc.
4	*F*5 + F15 + F16 + F18	10.54	SDDS	Tiredness and fatigue, spontaneous sweating, constant fear of cold, white tongue coating, greasy tongue coating, deep pulse, etc.
5	*F*4 + F10	6.90	PTUS	High fat diet, addicted to spicy food, smoking, blurred vision, dizziness, etc.

PHS: phlegm heat syndrome; HHFS: hyperactivity of heart fire syndrome; SDDS: spleen deficiency and dampness syndrome; DLKS: deficiency of liver and kidney syndrome; PTUS: phlegm turbid upper syndrome.

## Data Availability

The data used to support the findings of this study are available from the corresponding author upon request.
